# Dynamic spinal posture and pelvic position analysis using a rasterstereographic device

**DOI:** 10.1186/s13018-020-01825-0

**Published:** 2020-09-08

**Authors:** Roman Michalik, Juliane Hamm, Valentin Quack, Jörg Eschweiler, Matthias Gatz, Marcel Betsch

**Affiliations:** 1 Department of Orthopaedics and Trauma Surgery, University Hospital Cologne, Kerpener Str. 62, 50937 Cologne, Germany; 2 Department of Orthopaedics, University Hospital RWTH Aachen, Pauwelsstraße 30, 52074 Aachen, Germany; 3 Department of Anesthesiology, Heinrich Heine University Hospital Düsseldorf, Moorenstraße 5, 40225 Düsseldorf, Germany

**Keywords:** Surface topography, Spine, Dynamic, Gait posture, Rasterstereography

## Abstract

**Background:**

Until recently, rasterstereographic analysis of the spine was limited to static measurements. However, understanding and evaluating the motion of the spine under dynamic conditions is an important factor in the diagnosis and treatment of spinal pathologies. The aim of this study was to study the spinal posture and pelvic position under dynamic conditions and compare it to static measurements using a dynamic rasterstereographic system.

**Methods:**

A total of 121 healthy volunteers (56 females; 65 males) were included in this observational study. The parameters trunk inclination, trunk imbalance, pelvic obliquity, kyphotic angle, lordotic angle, surface rotation, and lateral deviation were studied and compared under static and dynamic (1, 2, 4, 5 km/h) conditions using the system “Formetric 4D Motion®“ (DIERS International GmbH, Germany).

**Results:**

Female volunteers had a higher lordotic angle than males under static conditions (*p* < 0.001). Trunk inclination (5.31° vs. 6.74°), vertebral kyphotic angle (42.53° vs. 39, 59°), and surface rotation (3.35° vs. 3.81°) increase under dynamic conditions (*p* < 0.001). Trunk inclination and lordotic angle both show significant changes during walking compared to static conditions (*p* < 0.001).

**Conclusion:**

The spinal posture differs between females and males during standing and during walking. Rasterstereography is a valuable tool for the dynamic evaluation of spinal posture and pelvic position, which can also be used to quantify motion in the spine and therefore it has the potential to improve the understanding and treatment of spinal pathologies.

**Trial registration:**

Retrospectively registered

## Introduction

Adolescent spinal scoliosis is a three-dimensional deformity of the spine, which is usually first diagnosed between the ages of 10–16 years. The gold standard in diagnosis and follow-up of scoliosis are still whole spine radiographs. According to Nash et al. patients with scoliosis undergo up to 22 whole spine X-rays during a 3-year treatment period [1]. This may lead to an 8% higher cancer mortality rate in scoliosis patients, and a 4-times higher breast cancer risk in this population [2]. Due to the radiation burden of standard radiographs, radiation-free and non-invasive methods to measure spinal deformity have been developed early on. One of the first radiation-free devices is called the scoliometer, which was developed in 1984 by Bunnell [3]. This device can be used as a screening tool, since it allows to measure trunk asymmetry, which correlates with the amount of spinal deformity [3]. The first optical surface analysis system was based on Moiré-Topography, which uses an interference pattern that is projected onto the back surface of the patients. However, this method is technically challenging and the Cobb angle can only be estimated with this system.

In the 1980s the most comprehensive surface topography system (rasterstereography) was developed by Drerup and Hierholzer at the University of Muenster, Germany [4]. Over the years, rasterstereography has shown its high validity and reliability in numerous studies [5–7]. So far, rasterstereography was limited to static measurements of the spinal posture. However, the flexibility of the spine and the motion of its segments is an important factor for diagnosing and treating spinal pathologies, such as scoliosis. Because of that, rasterstereography has been continuously advanced from a static to a dynamic system. By using a 50 Hz digital video camera this technique can analyze the spinal posture and pelvic position during walking on a treadmill with velocities of up to 6 km/h. The images recorded by the camera serve as a basis to calculate a three-dimensional back surface map and subsequently a three-dimensional model of the underlying spine. In a previous study, Betsch et al. were able to demonstrate that the marker detection of this rasterstereographic device is within 1 mm under static and dynamic conditions, when compared to the gold standard in motion analysis, a VICON system [8]. Furthermore, Gipsman et al. demonstrated in their study that the average standard deviation of same-day repeated measurements of parameters like kyphosis, lordosis, and the rotation of pelvis were within ± 3° (range 0.51–2.3°) [9].

This is the first study to evaluate the spinal posture and pelvic position under dynamic conditions and compare it to static measurements of the spine using a dynamic rasterstereographic system.

## Material and methods

### Study group

A total of 121 volunteers (56 females; 65 males) were included in this study. Inclusion criteria were age over 18 years, ability to walk on a treadmill for a minimum of 90 s with a maximum speed of 5 km/h, as well as a body mass index of under 35 kg/m^2^. Excluded from this study were participants with musculoskeletal disorders, such as osteoarthritis and rheumatoid arthritis, any history of previous fractures as well as systemic diseases that could potentially influence walking on a treadmill. All participants gave their oral and written consent to participate in this study and were given the option to discontinue their participation at any time. The local human ethics committee of the institute approved the protocol of the study (study number EK 3953).

### Rasterstereographic system

The “Formetric 4D Motion®“ system (DIERS International GmbH, Germany) is based on the method of stereophotogrammetric surface measurements of the back [8]. It uses a slide projector to project horizontal parallel light lines onto the back surface of patients. Then, an image or video is taken of the back surface with a digital network camera at 50 Hz. This camera uses a CMOS sensor with a resolution of 1280 × 1024 pixels. The software performs a digital reconstruction of the back surface by transforming the horizontal light lines and their corresponding curvatures into a three-dimensional scatter plot. Based on the specific shape of the spinous processes of the vertebra prominence (VP) and the concavity of the lumbar dimples, as anatomical reference points, a model of the spine is created. With this technique, transverse, axial, and sagittal profiles as well as several spinal and pelvic angles can be calculated. In contrast to other surface topography systems, rasterstereography allows an analysis not only of the back surface, but also of the underlying spine. This is made possible by using a spine model created by Drerup and Turner-Smith, based on more than 500 whole spine radiographs of patients with scoliosis [8, 10]. Due to movements of the skin and soft tissues above the anatomical landmarks during motion it is necessary to use infrared reflecting markers on the VP and the two lumbar dimples. In a previous study, Betsch et al. were able to demonstrate that the dynamic rastersterographic system is able to detect these markers with an accuracy of ± 1 mm under static and dynamic conditions using an array of 8 LEDs [8]. The position of the markers can be automatically detected with the use of an algorithm that scans the back surface for all bright elliptical regions. Then, from the position of the markers, a sub-pixel approximation of the center of the marker is used to determine its exact position. In approximately 100 ms, a complete reconstruction of the back surface is possible with these algorithms, making a real-time display of the spine and back surface during dynamic measurements possible.

### Measuring setup

All volunteers were measured in shorts and women were offered a drape to cover their chest so that it would not be visible from the back. Care was taken to provide free views on the VP and the two lumbar dimples. Three flat adhesive markers were placed on the back surface of the volunteers, one on the VP and two on the lumbar dimples, by the examiner. To verify the correct marker position, we performed a static scan of the back surface, and if necessary, the position of the marker was corrected. Once the correct placement of the marker was confirmed, they were kept in place for the following dynamic measurements.

### Measuring protocol

After confirmation of the correct marker placement a total of three static measurements were conducted. For all static measurements, the volunteers were placed on a treadmill with a distance of 2 m from the camera. All volunteers were instructed to stand in the neutral standing position with their arms hanging to the sides and extended knees. All dynamic measurements were conducted while the volunteers were walking on a treadmill to ensure an approximate distance to the camera of about 2 m. The subjects were measured three times while walking with velocities of 1, 2, 4, and 5 km/h. On average, all volunteers walked for 60 s on the treadmill to adjust to the treadmill velocity. The subsequent dynamic rasterstereographic recording lasted for 6 s, which resulted in 330 to 335 frames per measurement. After the recording, the treadmill was automatically stopped by the system and the software processed the collected data, while the subjects were resting for 2 min before the next trial.

### Data analysis

All parameters analyzed in this study are defined in Table 1. The average values of all three static and dynamic trials, including all four different velocities, were calculated by the examiner. Based on these values, we calculated the standard deviations for all parameters and conditions to be able to compare static with dynamic and all dynamic measurements.

**Table 1:** Measured parameters under static and dynamic conditions

Trunk length [mm]	Distance from C7 to the center between the lumbar dimples
Trunk inclination [°]	Angle between the defined trunk length and an external vertical line (positive angle = inclination; negative angle = reclination)
Trunk imbalance [°]	Lateral deviation of VP from the centre between the lumbar dimples (positive angle = VP is shifted to the right related to midpoint of the lumbar dimples)
Pelvic obliquity [°]	Angle between the lumbar dimples and a horizontal line. (positive angle = right lumbar dimple is higher than the left one)
Kyphotic angle [°]	Angle between the superficial tangent of the spinous process of C7 and the calculated spinous process of T12
Lordotic angle [°]	Angle between the superficial tangent of the spinous process of T12 to the midpoint of the lumbar dimples
Surface rotation [°]	The root mean square between the angles of the horizontal components of the normal surface lines of the symmetric lines and the frontal plane
Lateral deviation [mm]	The root mean square between the distance of hull length and the center line of the vertebral column

### Statistical analysis

All data were processed and prepared by the programme “Dicam II 2.2.3” (DIERS International GmbH, Germany). SPSS Statistics Version 20 was used for all statistical analysis (IBM, USA). The differences in the anthropometric data and static parameters between male and female participants were evaluated with the Mann-Whitney *U* test (level of significance α < 0.05). As the Kolmogorov-Smirnov test revealed no normal distribution for any data in this study, we used non-parametric statistical tests. A Kruskal-Wallis test was used to analyze the parameters collected during different walking speeds for significant differences. If so, significance was verified by using a Mann-Whitney *U* test. Furthermore, the level of significance was corrected by using Bonferroni correction at a significance level of *p* < 0.0083 for analyzing data during different walking speeds. For comparing dynamic with static parameters, the same procedure was used, with a Bonferroni correction at a significance level of *p* < 0.0125.

## Results

The anthropometric data of the volunteers is summarized in Table 2. Female participants were significantly younger, smaller, and lighter than males (Table 2) (*p* 0.036–0.001).

**Table 2:** Anthropometric data of the study group divided by sex (total *n* = 121, female *n* = 56, male *n* = 65). Significant differences between females and males are in bold

	Females (*n* = 56)		Males (*n* = 65)		Total (*n* = 121)		Mann-Whitney *U*		
	Mean	SD	Mean	SD	Mean	SD	*u*	*z*	*p*
Age [a]	23.57	2.0	24.29	2.22	24.07	2.16	1421.5	− 2.098	**0.036**
Height[cm]	168.91	6.38	182.52	5.78	176.22	9.1	213.5	− 8.358	**< 0.001**
Weight [kg]	62.89	8.5	79.91	8.97	72.03	12.19	256.5	− 8.135	**< 0.001**
BMI [kg/m^2^]	22.01	2.43	23.98	2.43	23.07	2.61	950.5	− 4.521	**< 0.001**

### Comparison of static measurements female vs. male

As shown in Table 3, women were significantly (Mann-Whitney *U*: *U* = 820.5, *p* < 0.001) smaller than men with a mean (±SD) trunk length of 452.38 (±26.72) vs. 492.82 (±28.43). Furthermore, female volunteers had a significantly (Mann-Whitney *U*: *U* = 524, *p* < 0.001) greater lordotic angle (37.36°± 9.79 vs. 28.96 ± 7.67) as men. For all other parameters, we did not find statistical differences between women and men.

**Table 3:** Static and dynamic measurements: mean and SD of all parameters of female, male, and the whole study population (static and 1–4 km/h: *n* = 121, female = 56, male = 65; 5 km/h *n* = 110, female = 51, male = 59)

	(km/h)	Females		Males		Total	
		Mean	SD	Mean	SD	Mean	SD
Trunk length [mm]	Static	452,38	26,72	492,82	28,43	474,11	34,18
	1	456,98	25,44	496,82	26,64	478,38	32,76
	2	458,70	25,24	497,46	26,82	479,52	32,44
	4	459,84	26,10	498,26	26,85	480,48	32,66
	5	457,85	27,21	496,72	28,09	478,70	33,75
Trunk inclination [°]	Static	2.12	2.40	1.89	1.88	2.00	2.13
	1	5.01	2.33	5.57	1.81	5.31	2.08
	2	5.52	2.39	5.86	1.84	5.70	2.11
	4	6.11	2.46	6.36	1.92	6.24	2.18
	5	6.72	2.62	6.76	2.24	6.74	2.41
Trunk imbalance [°]	Static	− 0.07	0.91	− 0.08	0.96	− 0.08	0.93
	1	− 0.20	0.82	− 0.15	0.96	− 0.18	0.89
	2	− 0.19	0.88	− 0.16	0.87	− 0.17	0.87
	4	− 0.31	0.94	− 0.21	0.90	− 0.25	0.92
	5	− 0.27	1.00	− 0.17	0.86	− 0.22	0.92
Pelvic obliquity [°]	Static	− 0.42	2.79	− 0.32	3.34	− 0.37	3.09
	1	− 0.24	2.16	− 0.45	2.78	− 0.35	2.50
	2	− 0.24	2.08	− 0.37	2.73	− 0.31	2.44
	4	− 0.28	2.02	− 0.34	2.62	− 0.31	2.35
	5	− 0.29	2.04	− 0.51	2.61	− 0.41	2.36
Kyphotic angle [°]	Static	44.02	8.64	44.58	7.84	44.32	8.19
	1	42.30	7.90	42.72	7.09	42.53	7.44
	2	41.16	7.99	41.79	7.21	41.50	7.55
	4	40.15	8.35	40.70	7.26	40.45	7.76
	5	39.27	8.51	39.86	7.72	39.59	8.06
Lordotic angle [°]	Static	37.36	9.79	28.96	7.67	32.85	9.64
	1	33.64	7.17	24.34	6.95	28.64	8.43
	2	31.94	7.43	23.31	7.08	27.30	8.41
	4	30.17	8.39	21.94	7.52	25.75	8.91
	5	30.29	8.82	21.91	7.67	25.80	9.20
Surface rotation [°]	Static	3.64	1.62	3.54	1.56	3.59	1.59
	1	3.49	1.11	3.24	0.98	3.35	1.05
	2	3.46	1.12	3.19	1.03	3.32	1.08
	4	3.78	1.05	3.26	0.95	3.50	1.02
	5	4.23	1.03	3.45	0.93	3.81	1.05
Lateral deviation [mm]	Static	5.59	2.32	5.07	2.13	5.31	2.23
	1	4.69	1.90	4.64	1.21	4.66	1.56
	2	4.74	1.79	4.56	1.23	4.64	1.51
	4	5.20	1.78	4.41	1.22	4.77	1.55
	5	5.52	1.72	4.60	1.30	5.03	1.58

### Comparison of dynamic measurements female vs. male

We did find intersexual significant differences (*p* < 0.001) for trunk length and lordotic angle for all measured walking velocities (1, 2, 4, and 5 km/h). In summary, women had a greater lordotic angle while walking on a treadmill. Interestingly, women also had significant greater values of surface rotation and lateral deviation than men; however, only for increasing walking velocities of 4 km/h (*p* = 0.004) and 5 km/h (*p* < 0.001). For all other parameters, we did not find significant intersexual differences for all walking velocities (*p* > 0.05).

### Comparison of dynamic measurements with different walking velocities

First, we evaluated and compared the trunk inclination between the different walking velocities. With increasing walking speed, we did find an increase in trunk inclination between 1 vs. 4 km/h, 1 vs. 5 km/h, and between 2 vs. 5 km/h (*p* < 0.001). We also measured a decrease in the kyphotic angle with increasing walking speed, which was statistically smaller between 1 vs. 5 km/h (*p* < 0.001). No significant differences were found in our study for the lordotic angle and lateral deviation, which did not change with increasing walking speed (*p* > 0.05). A parameter that measures the rotation of the spinous processes is called surface rotation. The surface rotation also increased significantly with increasing walking velocities, 1 vs. 5 km/h (*p* < 0.001) as well as 2 vs. 5 km/h (*p* < 0.007).

### Comparison of dynamic with static measurements

**Fig. 1:**
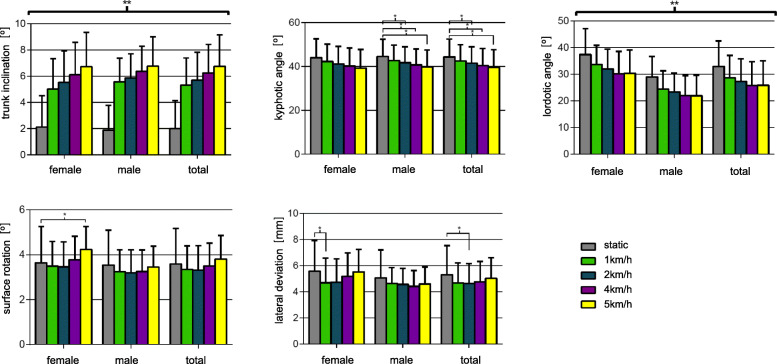
Dynamic measurements of trunk inclination [°], kyphotic angle [°], lordotic angle [°], surface rotation [°], lateral deviation [mm]: comparison between static and dynamic measurements (static, 1 km/h, 2 km/h, 4 km/h: total *n* = 121, females *n* = 56, males *n* = 65 error bars ±1 SD; *Significant *p* < 0.0125)

To evaluate if differences of the parameters occur between standing in the neutral position and while walking on a treadmill, we compared static versus dynamic measurements (Fig. 1). For the trunk inclination, we did find significant differences between the static condition and all four walking velocities (*p* < 0.001). There were also differences between the static measurements of the parameter kyphotic angle and walking speeds of 2 km/h (*p* = 0.003), 4 km/h (*p* < 0.001), and 5 km/h (*p* < 0.001). For the static condition of the lordotic angle, we did measure the highest value. We also found statistically lower lordotic angles with increasing walking velocities (*p* < 0.001). For the lateral deviation, we did only find a significant difference between the static measurements and the walking speed of 2 km/h (*p* = 0.0117). Finally, we did not find any differences between the static and dynamic measurements for the surface rotation (*p* = 0.039–0.800).

## Discussion

Spine X-rays are still considered the gold standard in diagnosing and treating spinal pathologies, although they result in a radiation burden for the patients [10]. A further disadvantage of X-rays is that they depend on the exact patient positioning, because minimal changes of their position can lead to systematic errors [11]. Rasterstereography is a fast, radiation-free, and objective alternative to X-rays with high reliability, which can help to minimize the need for repeat X-rays [12]. Due to continuous development, the rasterstereography system allows a dynamic analysis of the pelvic position and each of the spinal segments between C7 and L4. Dynamic measurements of the spine could help us in the future to better diagnose and understand the pathogenesis of spinal diseases, such as scoliosis or juvenile hyperkyphosis.

In a previous study, we were able to demonstrate that this novel rasterstereographic device can automatically detect flat, infrared marker on the back surface of patients with the same accuracy than a VICON system, which is currently considered the current gold standard in gait and motion analysis [8]. The marker detection was within 1 mm compared to the VICON system during static and dynamic measurements [8]. Motion analysis systems have mainly focused in the past on the examination and evaluation of the lower extremities. The back and trunk were mostly regarded as a single unit and their motion was evaluated in relation only to the motion of the pelvis [13, 14]. Due to technical limitations in the past, analysis of complex movements of the spine and its segments were not possible [15]. The aim of this study was to evaluate spinal posture under dynamic conditions by measuring 121 healthy volunteers. All volunteers were rasterstereographically scanned, and the results were then analyzed regarding their gender, condition (static versus dynamic), and walking speed (1, 2, 4, and 5 km/h).

Due to anthropometric reasons, men are taller than women in general, which was confirmed by gender specific differences in trunk length under static conditions and dynamic conditions. With increasing treadmill speed, we also found an increase in trunk length. Frigo et al. found a 3.5% increase in trunk length under dynamic conditions in comparison to the standing position. They explained their findings with lumbar straightening during walking by reducing the lumbar lordotic and thoracic kyphotic angle [14]. These results are in accordance to our findings, where we found a decrease in lordotic and kyphotic angles with increasing walking velocities.

During walking the spine is kept in a neutral orientation to minimize the motion between pelvis and the head [16]. This helps to stabilize our optical system and helps to improve orientation in space [17]. With this dynamic rasterstereography system, it is possible to quantify compensatory movements of the pelvis and spine during walking. The results of our study showed that with increasing walking velocities, there was a significant increase in trunk inclination, independent of the sex of the volunteers. These findings were confirmed in previous studies using different measuring systems [18–20]. The increase in trunk inclination with increasing walking speeds may be to balance the body during walking [16]. We also found a trend toward a decrease in kyphosis and lordosis with increasing walking speeds, which also results in an increase in trunk inclination as described above. This was also confirmed in various studies [14, 21, 22]. Drerup et al. found that the forward tilt of the trunk results in a decrease of the lordotic and kyphotic angles [21]. In our study, changes of the lordotic angle were more profound than of the kyphotic angle. The lordotic angle decreased between standing and walking with a speed of 5 km/h on average of 7.1°, while the kyphotic angle decreased only by 4.7°. We also measured the smallest kyphotic angle during walking with a speed of 5 km/h. In general, we did find a greater lordosis in females than in males, which was confirmed in multiple studies [23–25]. The surface rotation and lateral deviation of the spine are parameters that describe frontal changes of the spine. We did not find any gender differences for both parameters during standing. However, for walking speeds 4 and 5 km/h, there was a significantly greater surface rotation and lateral deviation measured for females than for males. So far, there do not exist any studies that have investigated this phenomenon, since most analysis methods cannot measure frontal spine parameters. A possible explanation for the gender differences could be differences of the stride length, which are significantly smaller in women. We also found differences between static and dynamic measurements for the surface rotation (SD 1.59° vs. 1.03°) and the lateral deviation (SD 2.23° vs. 1.59°). Further analysis of these parameters might be helpful in patients with frontal deformities of the spine such as scoliosis [26].

A limitation of this current study is the relatively small number examined (*n* = 121) and the close demographics of the volunteers. This makes it not feasible to analyze the data in regard to age and weight. Further studies with larger cohorts will be necessary to be able to compare the data for these variables and to be able to have reference values. A further limitation is that all volunteers were healthy and did not present with any spinal disorders. In future studies, we will have to measure patients with different spinal pathologies to be able to recognize pathology specific changes of the spine during motion. However, we believe it is necessary to first document and to understand the changes of the spine and pelvis measured with rasterstereography in healthy subjects before analyzing pathologies. So far, this rasterstereographic device was only able to measure minimum, maximum, and mean values of the parameters during a period of 6 s independent of the gait cycle of the subject. We believe that for a better understanding and comparison, these data need to be correlated to the gait cycle. Therefore, we are currently working on a software update that will enable us to measure all parameters in regard to the gait cycle as describe by Perry et al. [16].

## Conclusion

This is study measures and analyses dynamic rasterstereographic data of 121 volunteers. Our results did find differences between static and dynamic measurements that were comparable to the current literature. In addition, dynamic rasterstereography will help in the future to quantify motion in each spinal segment between C7 and L4 and in the pelvis, which potentially will lead to a better understanding of spinal pathologies.

## Data Availability

The data that support the findings of this study are available from Marcel Betsch and Juliana Hamm, but restrictions apply to the availability of these data, which were used under license for the current study, and so are not publicly available.
